# Syphilis and Life-Insurance

**Published:** 1904-07

**Authors:** 


### *EDITORIAL NOTES AND COMMENTS.*

The Business office of THE JOURNAL-RECORD is No. 1007-1008 Century Building.

The Editorial office is 318-319-320 The Prudential.

Address all Business communications to Dr. M. B. Hutchins, Mgr.

Make remittances payable to THE ATLANTA JOURNAL-RECORD OF MEDICINE.

On matters pertaining to the Editorial and Original Communications address Dr. Bernard Wolff, Atlanta.

Reprints of original articles will be furnished at cost price. Requests for the same should always be made *on the manuscript.*

We will present, post-paid, on request, to each contributor of an original article, twenty (20) marked copies of THE JOURNAL-RECORD containing such article.

### SYPHILIS AND LIFE INSURANCE.

IN an editorial the *Medical Examiner* calls attention to the small amount of consideration given by the medical examiner to the eventual presence of syphilis in an applicant. It is the universal experience of those who make insurance examination that a confession of past syphilis from the applicant is of the rarest. The impregnable virtue or the phenomenal luck becomes in this manner a conspicuous feature of American manhood. And yet syphilis has been and is one of the most frequent of the common diseases of mankind. To reconcile these facts with applicant's denial one must accept as explanation that unfailing resort of the hard-pressed—a lie, popular under similar circumstances since Adam. The examiner when he reaches this question and receives the usual negative answer accepts it without demur, whatever he may think of it. He rarely ever makes an investigation to establish the truth of the patient's statement by examining his person for stigmata of the disease. Yet of all the questions asked by the company there is assuredly none more important as concerns its interest than the one relating to syphilis. The condition of the patient's thoracic viscera, as well as some of the abdominal, can be oriented objectively with fair accuracy, but under the modern methods of therapy what remains to be seen in most cases of syphilis after 10 years, after 5 years, even after two years.

The prognosis of syphilis is a moot question, as every one knows. There are optimists, there are pessimists and there are individualists, but all unite in the belief that syphilis is irregular in its course and, like the bread on the water, may return after many days.

This prognostic uncertainty has materially influenced the policy of insurance companies in regard to accepting those who are frank enough to acknowledge a brush with syphilis. Some decline them altogether and some are guided by Fourrimer's dictum. But in any instance the risk is looked at askance and with misgiving. A partial way out of the difficulty lies not in attempting to improve the moral tone of the individual—for there is no hope for the liar—nor to endeavor to suppress the social evil, but the cultivation on the part of the medical examiner of caution and vigilance—caution in accepting an applicant's statement and vigilance in being on the alert for the hallmarks of the disease, which may sometimes be found though the person be as "virtuous as Joseph and as wise as Penelope." "And when found make a note of," to quote the favorite remark of the astute Captain Cuttle.

